# SARS-CoV-2 mRNA Vaccine Attitudes as Expressed in U.S. FDA Public Commentary: Need for a Public-Private Partnership in a Learning Immunization System

**DOI:** 10.3389/fpubh.2021.695807

**Published:** 2021-07-16

**Authors:** Elissa R. Weitzman, Amy C. Sherman, Ofer Levy

**Affiliations:** ^1^Division of Adolescent and Young Adult Medicine, Boston Children's Hospital, Boston, MA, United States; ^2^Center for Bioethics, Harvard Medical School, Boston, MA, United States; ^3^Precision Vaccines Program, Division of Infectious Diseases, Boston Children's Hospital, Boston, MA, United States; ^4^Department of Pediatrics, Harvard Medical School, Boston, MA, United States; ^5^Division of Infectious Diseases, Brigham and Women's Hospital, Boston, MA, United States; ^6^Broad Institute of the MIT and Harvard University, Cambridge, MA, United States

**Keywords:** COVID-19, mRNA vaccine, public attitudes, immunization policy and strategies, public commentary, ethics

## Abstract

As part of the U.S. Food and Drug Administration COVID-19 vaccine review process, public commentary was solicited offering an opportunity to reflect on vaccine attitudes that may impact the uptake of coronavirus vaccines. We identified themes in the commentary that highlighted the safety, efficacy, ethics, and trustworthiness and transparency regarding the novel mRNA COVID-19 vaccines. A “Learning Immunization System” model is proposed to optimize public, private, and academic partnerships relating to vaccine development and implementation.

## Introduction

After the U.S. Food and Drug Administration (FDA) Vaccines and Related Biological Products Advisory Committee (VRBPAC) meetings on 10 and 17 December, 2020, Emergency Use Authorizations (EUAs) were granted for *Pfizer BioNTech* (BNT162b2) and *Moderna* (mRNA-1273) vaccines. Reaching these milestones was historic and inspiring, yet acceptability of COVID-19 vaccines is not a given. Informed and clear clinical guidance, trusted spokespersons, transparency in business and science, and perceptions of personal risk drive vaccine acceptability (1, 2). These conditions may be especially important following EUA of novel vaccines, yet features of the COVID-19 pandemic work against them. Tensions exist within and between US federal agencies, the World Health Organization, and the private sector. Furthermore, the pandemic has been politicized, and dissemination of vaccine misinformation *via* social media is rampant (3, 4). Gross disparities in case counts across groups and nations coupled with poor health literacy amplify cynicism and reduce confidence (5). To further understand concerns regarding issuance of EUAs for the mRNA COVID-19 vaccines we reviewed public commentary submitted for the VRBPAC meetings. We sought to understand the areas of disconnection between the opportunity of vaccination to ameliorate the pandemic and hesitancy around vaccine rollouts under the EUAs. Themes that emerged from these public documents suggest the need for a model of unified collaboration between the public, vaccinologists and public health scientists, healthcare providers, pharmaceutical stakeholders, and policymakers. The model would enable a learning immunization development and deployment system that could be optimized from organizational, operational, and public communications perspectives to achieve public health goals.

## Vaccine Hesitancy in the Context of a Pandemic Response

In the history of vaccinology, typical timelines for vaccine development have been 10–20 years (6). In the context of a global pandemic, SARS-CoV-2 vaccines were developed under the *Operation Warp Speed* initiative within 9 months. U.S. national survey data find a substantial percentage (25%) of the population has little or no confidence in vaccine research and development; a larger percentage (39%) reports a great deal of confidence that scientists will act in the public's best interests (7). Though vaccine intentions and hesitancy are dynamic with varying estimates nationally and globally as of November 2020, 39% of respondents in a US national survey reported they will probably or definitely refuse SARS-CoV-2 immunization (7). The percentage of persons that needs to be vaccinated to achieve herd immunity is conditioned on many factors and is estimated to range from 67 to 90% (8). Notably the lower bound of these estimates is close to or exceeds the percentage of persons who report confidence and willingness to be vaccinated. Furthermore, vaccine confidence is closely linked to the perceived safety of vaccine products (9). Although mRNA vaccine technology has been studied for the past decade, it is a relatively new platform with no prior FDA approved mRNA vaccines on the market. Clearly, understanding and addressing concerns of the undecided and hesitant may support the sense of partnership needed to drive acceptability as SARS-CoV-2 vaccination campaigns continue (10).

## VRBPAC Public Commentary

To gain insight into the range of opinions regarding SARS-CoV-2 vaccines, we reviewed all public comments submitted to the VRBPAC in advance of EUA hearings for the *Pfizer* and *Moderna* vaccines. Commentary was solicited *via* the FDA website, Twitter, and announcements to FDA email lists. There were 860 comments entered from 27 November to 17 December 2020, nearly equal numbers for each meeting. Authors included citizens, trial participants, clinicians, other professionals (lawyers, clergy, physicians, scientists), and groups of persons including representatives of medical professional associations with memberships in the tens of thousands. The full corpus of each meeting docket was downloaded and read by one author (ERW), with co-authors (ACS, OL) reading portions, to identify main themes and subthemes. Of note, public comment review is not human subject research.

## Themes Identified Regarding Eua for Novel mRNA Vaccines

While multiple comments highlighted the importance of development of a SARS-CoV-2 vaccine and pressed the FDA for a speedy approval, the majority of comments expressed a range of concerns. The main themes that emerged for both vaccines reflected concerns about the **safety** of mRNA vaccines, **efficacy** in general and for subpopulations, **ethics** of plans for prioritizing groups for vaccination and adequacy of informed consent, protections from harm, rejection of vaccine mandates, and the **trustworthiness and transparency** of the pharmaceutical companies, government agencies, and regulatory processes. For each of these four themes, sub-themes emerged concerning adequacy and operations of vaccine clinical trials, post-authorization monitoring, public engagement, clinician education, and policy (Supplementary Table 1).

### Safety

Many individuals expressed concerns regarding safety of the mRNA vaccines, and rigor of safety criteria and assessments, especially in light of what was perceived to be a very rapid development of the mRNA vaccines under the U.S. government's *Operation Warp Speed*. A plurality of comments focused on the lack of information about the potential for side effects without longer-term follow-up and need to evaluate a broader range of endpoints. Concern was high regarding the potential for mRNA vaccines inducing disease and genetic changes. Aprehensions were expressed regarding special populations (e.g., individuals with prior COVID-19 disease, pregnant women, persons with chronic diseases, including allergies, autoimmune or immunocompromised conditions) who may have intrinsic characteristics that could affect the side effect profile. Questions were considerable around safety for persons of color (POC), presumed to be under-represented in the trials, with distrust reflecting awareness of present day and historical injustices.

### Efficacy

Concerns were voiced regarding efficacy across different populations and over time (long term durability), potential for virus transmission despite vaccination, and whether vaccination prevents asymptomatic infection. Validity standards for assessing efficacy were repeatedly questioned—how is efficacy measured accurately if the assays used keep changing? Threats to efficacy from poor measurement, reporting and sample biases, potential mismanagement of the cold chain were all raised. Concern was substantial around infrastructure and commitment to post-marketing surveillance to assess long term efficacy and safety. In addition, questions regarding efficacy for specific groups were prominent. Finally, questions were raised regarding the focus on vaccines vs. therapeutics or genetic testing of susceptibility for severe disease—often framed as a dichotomous choice between prevention and treatment.

### Ethics

Several issues regarding ethical principles were evident: (a) ongoing Phase III clinical trials, and (b) public distribution and use of a vaccine. For the clinical trials, many emphasized that trial participants should be unblinded and receive vaccination if initially in the placebo arm. Respect for participants and their assumption of risk was highly valued, with modest support for maintaining rigor, including through flexible trial designs (11). Emphatic cautionary messages were submitted that delays in unblinding would cause participants to exit the trials, with additional strong concern that this delay would also alienate the public from participating in research, jeopardizing future science. For the public, the comments highlighted concerns around vaccine mandates and violation of personal rights. For public distribution and use, significant concern was expressed about loss of agency and choice should vaccination be mandated—which was emphatically negatively viewed by a plurality of those who commented, with some notable exceptions that favored consideration of mandates for specific situations. Comments reflected concern for a slippery slope wherein a SARS-CoV-2 immunization mandate would open the door to mandates for preventive or screening medical procedures (e.g., mammograms and colonoscopies). Comments also reflected concern for fairness and equity and the need to prioritize vaccine distribution to medically vulnerable persons, and persons at higher risk of severe disease including POC.

### Trust and Transparency

The highly accelerated process for vaccine development was interpreted pejoratively and viewed as rushed and entangled with the U.S. political process, which undercut trust. Concern for commercialization, conflict of interest, and awareness of lobbying activities by the pharmaceutical industry reduced trust. Similarly, the process by which injury is asserted and compensated and the mechanisms by which accountability is established and liability protected against (no-fault compensation) were perceived as unclear and suspect (12). The no-fault protective mechanism that enables industry to take on risks to release vaccines under emergency use authorization was regarded by some as a means by which safety standards could be lowered, endangering the public. There was considerable worry about underrepresentation of POC and vulnerable populations in clinical trials, which can lead to mistrust and low uptake of the vaccine in these communities.

## Discussion

### A Vision for Coordination and Communication: Learning Immunization System

In sum, while the speed with which SARS-CoV-2 vaccines were developed was essential to combat the growing numbers of infections and deaths from COVID-19, the accelerated timeline generated a range of public reactions with acute relevance to vaccine acceptability in the areas of safety, efficacy, ethics, trust, and transparency. Concerns apply to the full arc of biomedical innovation—from the engineering process for material design and development, through testing and trialing, post-licensure monitoring, public engagement, clinician education, and policy development. Although volunteered commentary is not population representative nor summarized quantitatively given the focus on rapid thematic analysis, it may reveal the broad contours of issues impeding vaccine acceptability and guide outreach for successful vaccination campaigns. In reviewing commentary, it's notable that a substantial set of resources exist to address some concerns (Table 1). However, there seemed to be little awareness of these resources. Moreover, many people made it clear that important assets which might assuage doubt and direct action are missing—coordinated leadership, clarity, transparency, and opportunities for ongoing professional and public engagement to address (mis)understandings, questions, low confidence, and trust.

**Table 1:** Existing and needed infrastructure to target concerns about COVID-19 vaccinations.

	**Existing infrastructure**	**Additional infrastructure/tools needed**
**Vaccine clinical trials**		
	Phase III clinical trials: continued monitoring for efficacy, adverse events, and durability of immune response *via* adapted cross-over design	Dedicated clinical trials for special populations (e.g., pregnant women, immunocompromised, persons of color)
	Bridge trials for evaluation of vaccines in children	Additional studies for safety and efficacy (planned)
		Adapt current Phase III studies to evaluate additional outcome measures (e.g., transmission dynamics)
**Post-licensure monitoring**		
*General Public*	Vaccine adverse event reporting system, VAERS	Education regarding EUA v. licensure (BLA)
	Vaccine safety datalink, VSD	Public and clinician/provider education about monitoring tools and how to report
	Clinical immunization safety assessment project, CISA	Systems and monitoring tools for local pharmacies
	FDA biologics effectiveness and safety system, BEST	Engagement of technology sectors to implement easy reporting for vaccinated individuals/providers
	FDA and the Centers for Medicare and Medicaid Services: Medicare Data	Clear communication around accountability in the case of injury
	FDA: sentinel initiative	Transparency regarding funding for pharmaceuticals
	CDC V-safe: phone application	
*Acute Care and Long-term Care Facilities*	CDC National Healthcare Safety Network, NHSN	
*Military*	Department of defense VAERS	
	Department of defense vaccine adverse event clinical system, VAECS	
	Department of defense electronic health record and defense medical surveillance system	
*Veterans*	Veterans affairs adverse drug event reporting system, VA ADERS	
	Veterans affairs electronic health record and active surveillance system	
*Tribal Nations*	Indian Health Service, HIS VAERS	
**Public engagement**		
	CDC vaccine education pages (https://www.cdc.gov/vaccines/ed/patient-ed.html)	Sustained conversation with the community regarding vaccine progress and guideline updates that allows for a continuous iterative process and quality improvement.
	CDC frequently asked questions about COVID-19 vaccination	Forums for listening to public concerns on local levels (e.g., townhalls)
	Department of Health and Human Services COVID-19 Public Education Campaign	Vaccine education initiatives for persons of color who have experienced disproportionate disease burden
		Education regarding individual reporting of adverse events
**Clinician education**		
	CDC clinician vaccine education tools (https://www.cdc.gov/vaccines/ed/index.html)	Collated resource for updated information regarding vaccines
	COVID-19 Advisory Committee on immunization practices (ACIP) vaccine recommendations	Education for clinicians regarding vaccine adverse event reporting (what systems are available, how to report, etc.)
	Quality improvement projects that target immunization	Providers' experiences are used to inform evidence base
	CDC current issues in immunization webinar (https://www.cdc.gov/vaccines/ed/ciiw/index.html?CDC_AA_refVal=https%3A%2F%2F%2Fvaccines%2Fed%2Fciinc%2Findex.html)	Resources for specific populations (patients with comorbidities, persons of color, etc.) with updates as information becomes available. CDC directed with representation from specific medical societies.
	COVID-19 vaccine training: general overview of Immunization Best Practices for Healthcare Providers (https://www.cdc.gov/vaccines/ed/courses.html#covid-19)	Dissemination of specific information for targeted comorbidities or conditions through medical societies (e.g., American College of Obstetrics and Gynecology, American Academy of Pediatrics, etc.)
	Vaccinating pregnant and lactating patients against COVID-19 (ACOG)	
**Policy**		
	State recommendations regarding vaccination	National guidelines that address work place, school, and travel recommendations regarding immunization
	Local recommendations regarding vaccination	System that allows for critical curation of policies that govern roll-out of vaccination policies
	Institutional recommendations without standardization	System that allows for flexibility and adaption of policies as new information is acquired.
		Guidance around civil liberties with administration of EUA product

**Figure 1:**
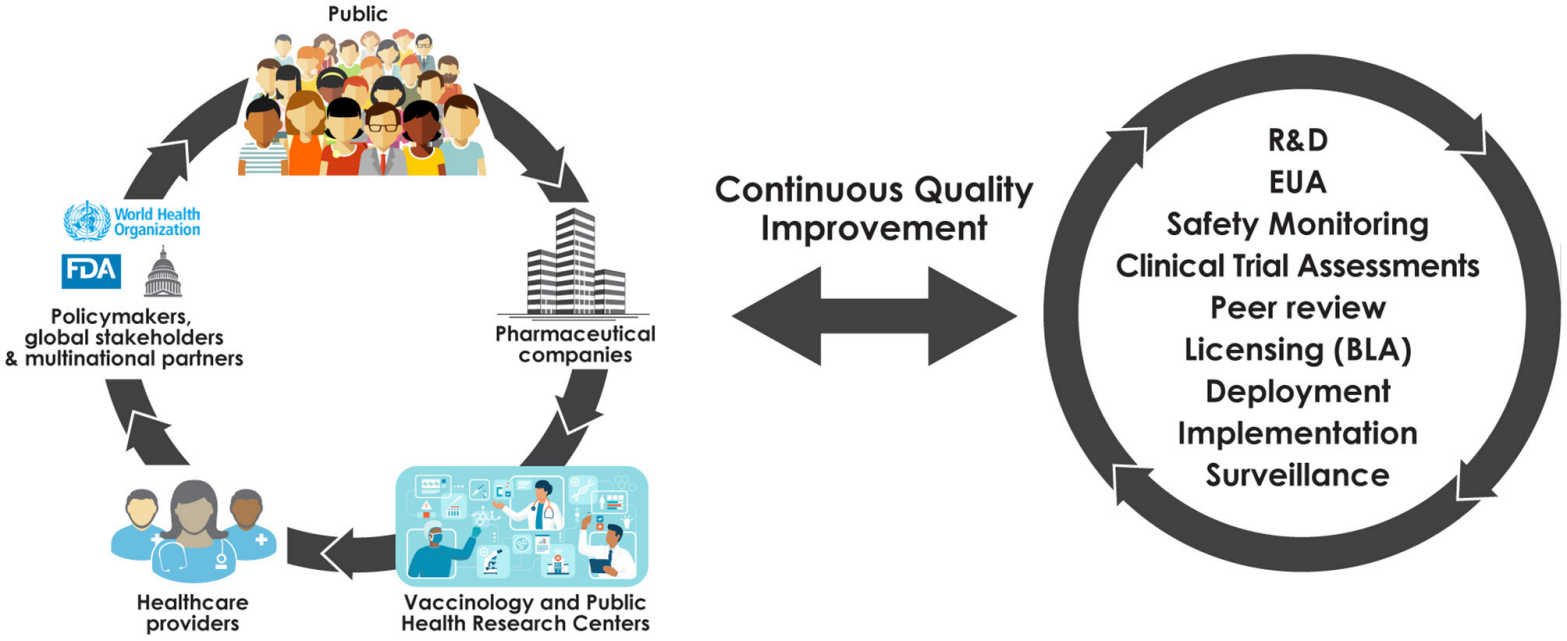
A learning immunization system.

Our observations suggest the need to rapidly bring together the collective energies found throughout the fragmented vaccine ecosystem. We propose a coordinated **Learning Immunization System** (LIS), a five-way partnership that engages the public, providers, vaccinology and public health research centers (e.g., as supported by intra- and extra-mural NIH funding), pharmaceutical stakeholders, and policymakers (Figure 1). Such a system, similar to the “learning health system (13),” would be comprised of representatives of the five groups and allow for communications between all involved parties, have transparent operations, a clear mechanism for supporting public engagement to advance vaccine acceptability and equipoise, and infrastructure sufficient to identify and respond to safety signals under conditions that maximize transparency. The LIS would build upon current resources and systems (Table 1) to enhance utilization of pre-existing services. Importantly, the LIS would also enable knowledge sharing to drive policy development as new information about vaccines is acquired and policies to manage supply and demand over a vaccine rollout are refined. Conversations among stakeholders through forums and public-facing town halls could be promoted, limiting the risk that evolving evidence sows confusion and distrust.

## Conclusions

The development of novel mRNA vaccines for COVID-19 and the subsequent EUAs for two effective and safe mRNA-based COVID-19 vaccines within 1 year of identifying the virus have been life-saving scientific and public health achievements. Additional types of coronavirus vaccines are under evaluation, such as the adenovirus-based vaccine from *Johnson & Johnson* that was recently provided an EUA. However, as the pandemic continues, it will be even more essential to ensure transparent communication and uptake of COVID-19 vaccines as we face additional challenges, including vaccination for pediatric populations (14) and other special populations, as well as the threat of emerging variant coronavirus strains. Stakeholders, including federal, state and local governments, health care providers, advocacy organizations and sponsors would do well to reflect on the themes raised through the mRNA SARS-CoV-2 vaccine FDA advisory meetings and to consider how these themes hold and evolve over time. Building trust will be paramount to successfully ending this pandemic, especially as new areas of uncertainty emerge and we look ahead to manage future pandemics and public health emergencies.

## Data Availability Statement

The raw data supporting the conclusions of this article will be made available by the authors, without undue reservation.

## Author Contributions

EW co-conceptualized the paper, reviewed all supporting materials, and wrote manuscript. AS contributed to conceptualizing the paper, materials review, and manuscript writing. OL co-conceptualized the paper, contributed to materials review, and manuscript writing. All authors contributed to the article and approved the submitted version.

## Conflict of Interest

OL is a named inventor on patents relating to anti-infectives, *in vitro* platforms that model human immunity, and vaccine adjuvants. He received a consulting fee from GSK in 2019. The remaining authors declare that the research was conducted in the absence of any commercial or financial relationships that could be construed as a potential conflict of interest.
