# Supplementary material for: SARS-CoV-2 mRNA Vaccine Attitudes as Expressed in U.S. FDA Public Commentary: Need for a Public-Private Partnership in a Learning Immunization System
Source: Front Public Health. 2021 Jul 16;9:695807. doi: 10.3389/fpubh.2021.695807 (PMC8322674; doi:10.3389/fpubh.2021.695807)
Supplement: Supplementary file 1 [file Table_1.DOCX]

**Supplemental Table 1:** Themes and issues discussed in public comments to the 10 and 17 December 2020 U.S. Food and Drug Administration (FDA) Vaccines and Related Biological Products Advisory Committee (VRBPAC) convened to consider an Emergency Use Authorization (EUA) for the *Pfizer-BioNTech* and *Moderna* SARS-CoV-2 mRNA vaccines

|  | **Safety** | **Efficacy** | **Ethics** | **Trust and Transparency** |
| --- | --- | --- | --- | --- |
| **Vaccine Clinical Trial** | Lack of safety data on long term effects, meaning of acute allergic reactions  Lack of safety data on mRNA technologies and questions about quality control and standards given speed  Safety concerns for special populations: people of color, people with co-morbidities, children/youth, elderly, women of childbearing age, pregnant and lactating women | Lack of data on how vaccines interrupt transmission  Vulnerable groups not represented  In the context of a pandemic with people dying, we have enough efficacy data from the trials, and there is an urgency to approve the vaccine | Prioritize groups (POC, others) at high risk / disease burden  Need expansive informed consent with ingredient disclosure  Need to unblind the clinical trials, don't penalize the participants | Vulnerable groups not represented  If participants in the placebo arm of the trials do not get unblinded and do not get the vaccine, threaten to exit trial, seek vaccine/antibody testing, reducing validity; public trust in participating in science reduced and will harm future science (no one will ever participate in another trial)  Multiple mentions of the history of unethical experimentation (Tuskegee trial), if don’t unblind and vaccinate placebo group, add to distrust and ethical injury |
| **Post-Licensure Monitoring** | Risk for sterility? Asthma? Autoimmune disorders?  Lack of safety data on long term effects, lack of understanding of likelihood and severity of possible side effects  Lack of safety data on mRNA technologies  Confusion regarding meaning of EUA  Concern about whether the vaccine could spread disease  Concerns about immediate side effects of mRNA vaccines (e.g. Bell’s palsy, allergic reactions) | Lack of data on how vaccines interrupt transmission  Need mechanisms for post market surveillance and infrastructure  Concern regarding efficacy, tracking and management if cold chain is compromised | Prioritize groups (POC, others) at high risk / disease burden  Need expansive informed consent with ingredient disclosure | Vulnerable groups not represented  Cannot evaluate safety/efficacy without knowing ingredients, access to more information including on ingredient |
| **Public Engagement** | Concern that vaccinated persons will relax personal protective behaviors (masking, distancing) or that others will do so, contributing to rise in infections  Seems like high side effects (mild, moderate)  “Natural” strategies and use of Vitamin C/D can combat COVID-19. Risk of vaccine side effects outweighs risks of COVID-19 with perception that COVID-19 has low fatality rates.  Parents/caregivers or individuals who are concerned because they have personally experience prior vaccine related injury | Need mechanisms for post market surveillance and infrastructure  Discussion that the mortality risk is small (<2%) and “natural” strategies are preferred especially in the context of risk/benefit for vaccine side effects  Need mechanisms for post market surveillance and infrastructure | Confusion about informed consent, risk/benefit  Challenges around weighing risk/benefit and interpreting safety signals make decision making difficult  Religious objections: immoral to use fetal tissue for creation of vaccines; vaccines disrupt the natural system that God created. | Inconsistent messaging, lack of clarity and understanding of ingredients, concern that social media discussions are being stifled and censored  Frustration around possible conflicts of interest  Concerns about “big pharma” and lack of accountability |
| **Clinician Education** | Seems like high side effects (mild, moderate)  Safety concerns for special populations: people of color, people with co-morbidities, children/youth, elderly |  | Health care providers cannot guide patients without better data or risk doing harm | Cannot evaluate safety/efficacy without knowing ingredients, access to more information including on ingredients |
| **Policy** | Concern that vaccinated persons will relax personal protective behaviors (masking, distancing) or that others will do so, contributing to rise in infections  Regulatory framework around no-fault compensation are confusing, "Catch 22" -- absent clear mechanisms, people confused/concerned for no recourse if injured and presence of these interpreted as a signal of likely risk and harm, undercuts trust. |  | Cannot mandate vaccination: it is coercive to condition travel, work, or ability to attend events on vaccination  Mandates are “un-American” | Regulatory framework around no-fault compensation are confusing, with weak regulatory control regarding pharmaceutical developments |
